# A recurrent, homozygous *EMC10* frameshift variant is associated with a syndrome of developmental delay with variable seizures and dysmorphic features

**DOI:** 10.1038/s41436-021-01097-x

**Published:** 2021-02-02

**Authors:** Diane D. Shao, Rachel Straussberg, Hind Ahmed, Amjad Khan, Songhai Tian, R. Sean Hill, Richard S. Smith, Amar J. Majmundar, Najim Ameziane, Jennifer E. Neil, Edward Yang, Amal Al Tenaiji, Saumya S. Jamuar, Thorsten M. Schlaeger, Muna Al-Saffar, Iris Hovel, Aisha Al-Shamsi, Lina Basel-Salmon, Achiya Z. Amir, Lariza M. Rento, Jiin Ying Lim, Indra Ganesan, Shirlee Shril, Gilad Evrony, A. James Barkovich, Peter Bauer, Friedhelm Hildebrandt, Min Dong, Guntram Borck, Christian Beetz, Lihadh Al-Gazali, Wafaa Eyaid, Christopher A. Walsh

**Affiliations:** 1 Department of Neurology, Boston Children’s Hospital, Boston, MA USA; 2 Division of Genetics and Genomics, Department of Pediatrics, Boston Children’s Hospital, Boston, MA USA; 3 Neurogenetics Clinic, Neurology Unit, Schneider Children Medical Center, Petah Tikvah, Israel; 4 Sackler School of Medicine, Tel Aviv University, Ramat Aviv, Israel; 5 Genetics Division, Department of Pediatrics, King Abdullah International Medical Research Centre, King Saud bin Abdulaziz University for Health Science, King Abdulaziz Medical City, Ministry of National Guard-Health Affairs (NGHA), Riyadh, Saudi Arabia; 6 Department of Urology, Boston Children’s Hospital, and Department of Surgery and Department of Microbiology, Harvard Medical School, Boston, MA USA; 7 Howard Hughes Medical Institute, Boston Children’s Hospital, Boston, MA USA; 8 Division of Nephrology, Department of Pediatrics, Boston Children’s Hospital, Boston, MA USA; 9 Centogene AG, Rostock, Germany; 10 Department of Radiology, Boston Children’s Hospital, Boston, MA USA; 11 Medical Institute of Medical Affairs, Sheikh Khalifa Medica City, Abu Dhabi, UAE; 12 Department of Pediatrics, KK Women’s and Children’s Hospital, Ramat Aviv, Israel; 13 SingHealth Duke-NUS Genomic Medicine Centre, Singapore, Singapore; 14 Stem Cell Program, Boston Children’s Hospital, Harvard Medical School, and Harvard Stem Cell Institute, Harvard University, Boston, MA USA; 15 Department of Pediatrics, United Arab Emirates University, Abu Dhabi, UAE; 16 Division of Genetics, Department of Pediatrics, Tawam Hospital, Al Ain, UAE; 17 Raphael Recanati Genetic Institute, Rabin Medical Center–Beilinson Hospital and Pediatric Genetics Clinic, Schneider Children’s Medical Center, and Felsenstein Medical Research Center, Petah Tikvah, Israel; 18 Pediatric Gastroenterology, Hepatology and Nutrition Clinic, Dana-Dwek Children’s Hospital, Tel Aviv Medical Center, Ramat Aviv, Israel; 19 New York University School of Medicine, Center for Human Genetics & Genomics, New York, NY USA; 20 Neuroradiology, University of California at San Francisco, San Francisco, CA USA; 21 Center for Rare Diseases (ZSE Ulm), Ulm University Medical Center, Ulm, Germany; 22 Department of Pediatrics, United Arab Emirates University, Al Ain, UAE; 23 Present Address: genetikum, Neu-Ulm, Germany

## Abstract

**Purpose:**

The endoplasmic reticulum membrane complex (EMC) is a highly conserved, multifunctional 10-protein complex related to membrane protein biology. In seven families, we identified 13 individuals with highly overlapping phenotypes who harbor a single identical homozygous frameshift variant in *EMC10*.

**Methods:**

Using exome, genome, and Sanger sequencing, a recurrent frameshift *EMC10* variant was identified in affected individuals in an international cohort of consanguineous families. Multiple families were independently identified and connected via Matchmaker Exchange and internal databases. We assessed the effect of the frameshift variant on *EMC10* RNA and protein expression and evaluated EMC10 expression in normal human brain tissue using immunohistochemistry.

**Results:**

A homozygous variant *EMC10* c.287delG (Refseq NM_206538.3, p.Gly96Alafs*9) segregated with affected individuals in each family, who exhibited a phenotypic spectrum of intellectual disability (ID) and global developmental delay (GDD), variable seizures and variable dysmorphic features (elongated face, curly hair, cubitus valgus, and arachnodactyly). The variant arose on two founder haplotypes and results in significantly reduced *EMC10* RNA expression and an unstable truncated EMC10 protein.

**Conclusion:**

We propose that a homozygous loss-of-function variant in *EMC10* causes a novel syndromic neurodevelopmental phenotype. Remarkably, the recurrent variant is likely the result of a hypermutable site and arose on distinct founder haplotypes.

## INTRODUCTION

The endoplasmic reticulum membrane complex (EMC) consists of multiple proteins that are highly conserved across eukaryotes.^1^ This complex has been shown to play a critical role as a transmembrane protein insertase, facilitating the proper insertion of some tail-anchored membrane proteins and multipass transmembrane proteins.^2,3^ Of the ten proteins that form the human EMC, only variants in *EMC1* have previously been associated with a genetic syndrome that includes global developmental delay (GDD), cerebellar atrophy, seizures, microcephaly, and vision abnormalities^4,5^ (OMIM 616875).

In this study, we report 13 individuals from seven consanguineous nuclear families who are affected with a syndromic phenotype including GDD, intellectual disability (ID), variable seizures, and variable dysmorphic features including a long face, curly hair, cubitus valgus, and arachnodactyly. This phenotype segregated with a homozygous *EMC10* frameshift variant that appears to be a mutational hotspot. Using in vitro studies, we provide additional evidence for the deleterious effect of this *EMC10* variant.

## MATERIALS AND METHODS

Clinical presentation was assessed by a clinical geneticist from one of the participating clinical centers, and informed consent for publication of individual photos was also obtained. Exome, genome sequencing, and/or single-nucleotide polymorphism (SNP) array, were performed either through clinical diagnostic testing at Centogene and/or through research settings. See Supplemental Material for institution-specific gene discovery methods. Collaborators were connected via Matchmaker Exchange^6^ and existing scientific networks. Genome-wide linkage analysis was performed to generate a logarithm of the odds (LOD) score from SNP array data using Merlin under a recessive mode of inheritance assuming a disease allele prevalence of 0.0001 and full penetrance. Haplotype analysis from exome and genome sequencing data considered only variants in regions that are covered by both genome and exome sequencing. A 2-Mb region up and downstream of the relevant *EMC10* variant was interrogated (chr19: 48981900–52961200 [hg19]) and filtered for high quality homozygous variants.

Further details of genetic and experimental methods can be found in Supplemental Material.

## RESULTS

### Genetic findings

**Fig. 1:**
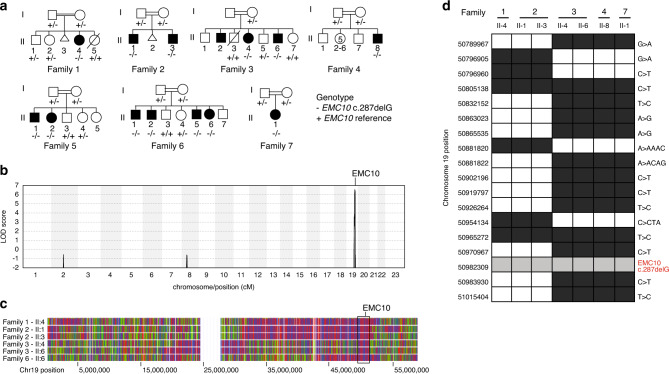
*EMC10* variant segregates with disease phenotype in multiple affected families. (**a**) Pedigrees of affected families. Affected individuals in families 1 and 2 are second cousins. Affected individuals in families 5 and 6 are first cousins. Solid black, affected. Genotypes, where indicated, represent results of evaluation for the *EMC10* c.287delG variant by Sanger sequencing. (**b**) Genome-wide logarithm of the odds (LOD) score distribution. (**c**) Affected individuals share a region of homozygosity on chromosome 19 (boxed), overlapping the location of *EMC10* variant. Single-nucleotide polymorphism (SNP) array data for affected individuals in families 1, 2, 3, and 6 are shown. Homozygous SNPs are displayed in red or blue. Heterozygous SNPs are displayed in green. (**d**) Haplotypes based on SNPs determined from sequencing data in the consensus region of homozygosity (chr19: 50789967–51015404) indicate that *EMC10* variant arose on two distinct haplotypes. SNP array independently confirmed two haplotypes (Fig. S2).

Using exome or genome sequencing, we identified a biallelic *EMC10* frameshift variant at RefSeq NM_206538.3: c.287delG (p.Gly96Alafs*9), in all affected individuals with shared phenotype in seven consanguineous families of Bedouin, Saudi Arabia, and United Arab Emirates origin (Fig. 1a). None of the individuals had other rare variants predicted to alter gene function in any previously reported genes associated with neurodevelopmental conditions. There are no individuals who are homozygous for the *EMC10* c.287delG variant in human reference databases such as gnomAD,^7^ GenomeAsia 100K Project,^8^ and the Greater Middle East Variome.^9^ No individuals with biallelic loss-of-function variants in *EMC10* were identified by comprehensively searching Centogene’s disease-associated variant database CentoMD,^10^ which contains data from >80,000 individuals with hereditary disorders analyzed by exome or genome sequencing. Sanger sequencing confirmed segregation of the *EMC10* variant with disease in all families, including the extended family tree of affected individuals in families 1 and 2 (Fig. S1).

Genome-wide linkage analysis to the phenotype of intellectual disability determined a maximum LOD score of 6.49 on chromosome 19, consistent with the location of *EMC10* (Fig. 1b). There were no other genomic regions that exhibited significant linkage. Linkage analysis using only array data for affected individuals (thus removing the assumption that siblings, who were not directly assessed, are truly unaffected) showed linkage to the same region. Regions of homozygosity (ROH) were also reviewed from SNP array, exome, or genome sequencing data, and ranged from 1.1 Mb to 2.8 Mb. The consensus ROH was ~225 kB in size (hg19 chr19: 50789967–51015404), in which the only shared coding variant was in *EMC10* (Fig. 1c).

Because the variant was identical in multiple unrelated families, we looked specifically at whether there was a founder effect (i.e., a single shared haplotype) or a potential hotspot for genetic variation (i.e., a variant that arose in multiple haplotypes). Haplotype analysis clearly showed two distinct haplotypes based on SNPs from exome sequencing (Fig. 1d). SNP array data also supported the presence of two distinct haplotypes, and included family 6 for whom exome data was unavailable (Fig. S2). Haplotypes were shared by affected individuals of families 1 and 2 who are second cousins, and a separate haplotype was identified in families 3 through 7.

### Clinical characteristics

**Fig. 2:**
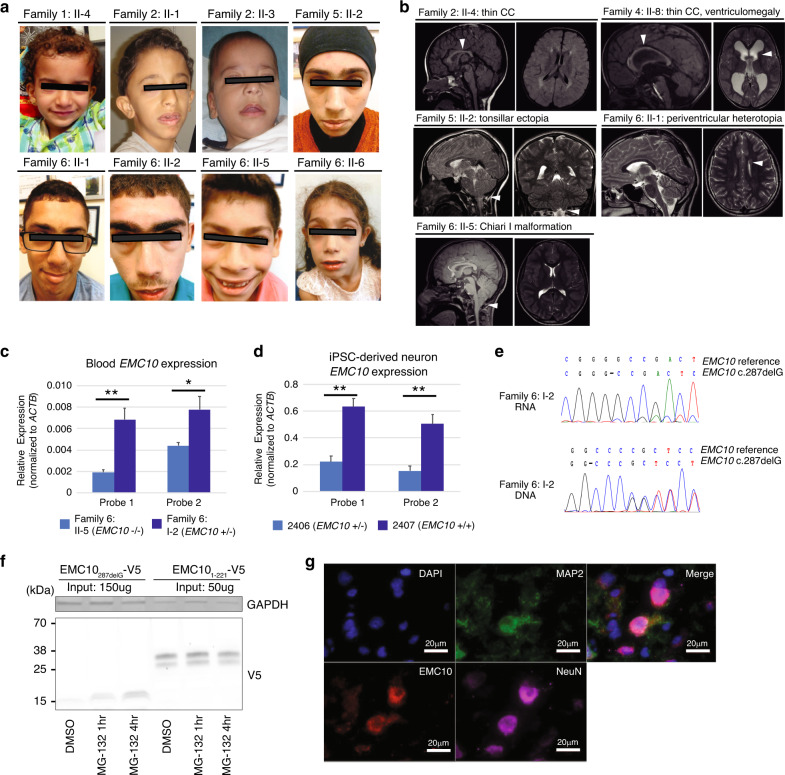
Clinical features of affected individuals. (**a**) Facial appearance of affected individuals. (**b**) Representative brain abnormalities on magnetic resonance image (MRI). See Table S2 for summary of imaging findings. (**c**) *EMC10* RNA expression relative to *ACTB* in blood from affected individual and unaffected parent. Single-sided *t*-test *p < 0.01, **p < 0.005. (**d**) *EMC10* RNA expression relative to *ACTB* in induced pluripotent stem cells (iPSC)-derived neurons from individual heterozygous for *EMC10* variant, and related individual without the variant. Both individuals are relatives of family 2 (pedigree in Fig. S1). Single-sided *t*-test **p < 0.005. (**e**) Sanger sequencing traces from DNA and RNA (complementary DNA [cDNA]) from an individual heterozygous for the reported *EMC10* variant. The DNA sequencing trace is displayed as reverse complement. (**f**) Proteasome inhibition by MG-132 rescues expression of V5-tagged truncated EMC10_287delG_ in a time-dependent manner. (**g**) EMC10 protein (red) is observed primarily in neurons in primary tissue from human brain. Neurons are marked by nuclear membrane staining for NeuN (magenta) or non-nuclear staining for MAP2 (green). EMC10 colocalizes with neuronal markers MAP2 and NeuN.

Clinical findings for all 13 affected individuals show a core phenotype of GDD/ID and, to a lesser extent, dysmorphic features and seizures (Table S1). Facial dysmorphisms described in multiple individuals include a long face, pointed chin, and curly hair, although evaluation by several dysmorphologists did not concur on a consistent facial gestalt (Fig. 2a). Limb anomalies included cubitus valgus (6/13), arachnodactyly (3/13), and bilateral 5th digit clinodactyly (1/13). Most individuals exhibited GDD in domains including social, motor, language, and cognitive, and/or ID (11/12). Individual II-1 in family 7 was age 3 months at ascertainment; thus most milestones could not be assessed. Seizures were noted in 6/13 individuals, typically during childhood or in the neonatal period, and included multifocal as well as generalized tonic–clonic seizures. The majority of affected individuals who underwent brain magnetic resonance imaging (MRI) had abnormal findings (9/10); however, findings were individually nonspecific, including cerebellar tonsillar ectopia or Chiari I (4/12), a thin corpus callosum (3/10), and white matter signal abnormalities (3/10) (Fig. 2b; Table S2). Neurologic symptoms appeared to be static or nonprogressive.

Additional minor features included failure to thrive (4/13), umbilical and inguinal hernias (5/13), and ventricular septal defects (2/13). Renal abnormalities of any kind were present in 9/13 affected individuals (69%) (Fig. S3; Table S3). There is no correspondence of renal phenotype to the two haplotypes at the *EMC10* locus. Renal abnormalities included nephrocalcinosis (4/11), mild hydronephrosis or hydroureter (2/11), and renal cysts (3/11; unilateral cyst in 2 individuals, bilateral cysts in 1 individual). One individual had end-stage renal disease of unclear etiology, which required kidney transplantation. The variability in renal phenotype was suggestive of different underlying genetic mechanisms, and less likely to be attributed specifically to the *EMC10* variant. Multiple liver cystic lesions were incidentally identified individual II-6 from family 6 (Fig. S4).

### *EMC10* functional assessment of variant and expression in human brain tissue

The *EMC10* c.287delG frameshift variant is expected to result in nonsense-mediated messenger RNA (mRNA) decay. *EMC10* expression is significantly reduced, but not absent, in affected individuals as determined by evaluation of RNA expression from blood by droplet digital polymerase chain reaction (PCR) in family 6: II-5 and his unaffected mother I-2, who is heterozygous for the *EMC10* variant (Fig. 2c). For each sample described, a negative control reaction performed without reverse transcriptase confirmed that there was no DNA contamination in the extracted RNA. Furthermore, in neurons derived from induced pluripotent stem cells from relatives of families 1 and 2 (individuals 2406 and 2407 indicated in Fig. S1; Fig. 2d), *EMC10* expression is reduced in heterozygotes compared with individuals without the variant. Finally, we amplified EMC10 complementary DNA (cDNA) in family 6: I-2 (*EMC10* + /-) using PCR, and showed that Sanger sequencing traces confirmed allelic imbalance, with decreased abundance of RNA transcripts that harbor the single-nucleotide deletion (Fig. 2e).

Although a small fraction of RNA that includes the *EMC10* frameshift variant is expressed, we show that this transcript results in an unstable protein. The potential truncated protein is 103 amino acids in length. The last 8 amino acids are altered due to frameshift and disrupts a region of high amino acid conservation (Figs. S5, S6). A signal peptide (amino acids 1–27) is cleaved in the mature form of EMC10 (UniprotKB accession U5QCC4). The C-terminal region, which interacts with core EMC proteins,^11,12^ would be abolished by the truncation. We cloned the open reading frame of *EMC10*, from residue 1 to the terminal residue of p.Gly96Argfs*9, into an expression vector (EMC10_287delG_). We also created another EMC10 truncation mutant that stops at residue 103 (EMC10_1–103_), which retains the wild-type amino acids but mimics the length of the truncation variant. Finally, we created a truncated 221 residue construct that included the entire lumenal domain (EMC10_1–221_), as control for expression of our allele. All constructs were tagged with a V5 epitope for detection and expressed in HeLa cells via transient transfection (Fig. S7). EMC10_1–221_ was detected in cells; in contrast, neither EMC10_287delG_ nor EMC10_1–103_ was detectable in cell lysates, suggesting that these two shorter truncated fragments are unstable. We showed that this instability is due to proteasomal degradation. Cells transfected with the EMC10_287delG_ or EMC10_1–221_ construct were treated with proteasomal inhibitor MG-132, which rescued protein expression in a time-dependent manner (Fig. 2f).

We assessed expression of endogenous EMC10 in postmortem infant human brain using immunohistochemistry (Fig. 2g). Specificity of the EMC10 antibody was confirmed by immunoblotting for EMC10 after transfection of commercially validated small interfering RNA (siRNA) (Fig. S8). Staining for EMC10 in postmortem human infant brain showed colocalization with MAP2, a non-nuclear protein expressed in mature neurons. NeuN, a nuclear marker of mature neurons, also showed colocalization with EMC10.

## DISCUSSION

We describe a syndromic phenotype including GDD/ID, seizures, and variable dysmorphic features and limb abnormalities, associated with autosomal recessive inheritance of a recurrent, loss-of-function frameshift variant in *EMC10*. Our cohort exhibits multiple renal abnormalities, which are difficult to reconcile with a single underlying genetic mechanism; thus, we cannot confidently ascribe a renal phenotype to the reported variant. Intriguingly, all the families identified shared the exact same *EMC10* variant, and we showed that the variant arose independently in two founder haplotypes. The single-nucleotide deletion occurs in a homopolymeric repeat sequence (CGGGGC) that predisposes to DNA replication errors and represents a potential hotspot for genetic variation. In addition, deletion of any one of the four consecutive G residues would create an indistinguishable frameshift allele. Recurrent variants at sites of homopolymeric G/C nucleotides have been identified in several disorders with monoallelic^13,14^ or biallelic^15^ inheritance.

Comparison of the *EMC10* phenotype to the published *EMC1* phenotype^4^ shows common features of GDD, present in all families for both diseases (Table S4). Individuals with *EMC10* variants had higher rates of seizures compared to *EMC1*, and individuals with *EMC1* variants had cerebellar or cerebral atrophy that was not seen in the *EMC10* cohort. Abnormalities of the corpus callosum were observed in both cohorts.

Homozygous *EMC10* knockout mice were characterized by the International Mouse Genotyping Consortium^16^ (www.mousephenotype.org) and exhibited statistically significant changes on behavioral assessments compared with control mice. Differences include abnormal vocalization, gait, activity, and behavior during open field testing, which measures motor activity and anxiety-related behaviors in rodents (Fig. S9). Another *EMC10* knockout model identified differences in cognitive processes such as working memory and associative emotional learning.^17^ Other published murine knockout models did not specifically assess neurobehavioral phenotypes.^18,19^ Further studies are required to understand whether the observed abnormalities in murine models directly reflect the neurocognitive profiles of humans with *EMC10* variants.

Transmembrane proteins have a spontaneous rate of protein membrane insertion, and this rate is enhanced by a functioning EMC.^2^ Changes in EMC function are likely to decrease, but not abolish, the insertion and proper function of multiple transmembrane proteins. Indeed, a survey of 61 proteins dependent upon the EMC in proteomic studies (Tian et al.^20^ and Shurtleff et al.^21^) revealed that many have been independently implicated in human neurodevelopmental diseases (Table S5). EMC10 is ubiquitously expressed in the body including in the brain, kidney, gastrointestinal tract, and musculoskeletal tissue;^22^ thus it is not surprising that the disease phenotype also involves multiple organs.

In summary, we implicate *EMC10* as a gene whose disruption leads to a human neurodevelopmental syndrome. The systemic nature of the phenotype highlights the pleiotropic roles of the EMC. Open questions remain in terms of the variability of the phenotype despite a single recurrent variant and the functional role of EMC10 in different organ systems.

## Supplementary information

Supplementary Information

## Data Availability

De-identified materials, data sets, and protocols are available upon request. The reported variant was submitted to ClinVar: SUB8682021.
