# Supplementary material for: A recurrent, homozygous EMC10 frameshift variant is associated with a syndrome of developmental delay with variable seizures and dysmorphic features
Source: Genet Med. 2021 Feb 2;23(6):1158–62. doi: 10.1038/s41436-021-01097-x (PMC8187145; doi:10.1038/s41436-021-01097-x)
Supplement: Supplementary file 1 — Supplementary Information [file 41436_2021_1097_MOESM1_ESM.pdf]

Supplementary Methods

Identification of EMC10

Families 1, 2, 3, 4, 7 were identified through clinical diagnostic exome sequencing performed at Centogene. In step 1 of Centogene's routine diagnostic workflow, variants in all OMIM genes are considered. If such variants do not fully explain the patient's phenotype, non-OMIM genes are considered in a subsequent analytical step 2. For all described patients, step 1 was negative, while step 2 flagged the reported *EMC10* variant.

Families 1, 2, and 3 were identified by research efforts at Boston Children's Hospital. In Family 3, the reported EMC10 was the only homozygous loss-of-function ultrarare variant shared between the 2 affected individuals (gnomAD allele fraction <0.0001). Family 1 was found to have the same *EMC10* variant on exome sequencing, as well as affected relatives (Family 2) sequenced at Centogene. SNP array and autozygosity mapping further supported linkage of the phenotype to chromosome 19 locus including EMC10 in all families.

Families 5 and 6 (affected individuals are first cousins) were identified by research efforts at University of Ulm. SNP array and autozygosity mapping identified a region of linkage at hg19 chr19:49519466-51004754. Family 6: II-1 had exome sequencing. There were no predicted loss-of-functions variants in the linked region except for the reported variant in *EMC10*.

DNA Sequencing and Analysis

Exome or genome sequencing by collaborating centers was performed either in the setting of clinical diagnostic testing through Centogene or through research settings at University of Ulm, Yale Genomics Center, the Broad Institute, or Beijing Genomics Institute (BGI) (Table S6; Table S7). Data for all institutions were aligned to the Human Genome Browser data, hg19 assembly (http://www.genome.ucsc.edu). Centogene performed WGS on family 1, 2, and 4, all other individuals sequenced at Centogene and other sites performed

exome sequencing. Details of Centogene-specific sequencing methods and variant calling have been previously described in Trujillano et al. (*Eur J Hum Genet* 2017) and Bauer et al. (*Genet Med* 2019). Broad Institute exome sequencing was performed using Agilent Sure-Select Human All Exon v2.0 capture array (Thermo Fisher Scientific) and sequenced on Illumina HiSeq2000 sequencers; BGI sequencing was performed using Agilent Sure-Select Human All Exome 37Mb V1 kit and sequenced on Illumina HiSeq2000 sequencers; Variant analysis from sequencing performed at the Broad Institute and BGI were performed at the Broad Institute using GATK best practices. Sequencing at Yale genomics was performed using Agilent SureSelect™ human exome capture arrays (Thermo Fisher Scientific) with next generation sequencing (NGS) on an Illumina™ platform. For WES data from Yale, sequence reads were mapped against the human reference genome (NCBI build 37/hg19) using CLC Genomics Workbench (version 6.5.1) (CLC bio). Variant calling was done using GATK5 in line with proposed guidelines (MacArthur, *et al. Nature* 2014: 508, 469) and criteria for determining candidate genes were employed as previously described (Vivante, A & Hildebrandt F, Nat Rev Nephrol 2016: 12, 133)(van der Ven et al., J Am Soc Nephrol 2018: 29, 2348). For exome sequencing at University of Ulm, genomic DNA was enriched for exonic and adjacent splice site sequences with the SeqCap EZ human exome library v2.0 kit, and libraries were run on an Illumina HiSeq 2000 sequencer via a paired-end 100-bp protocol. Variant analysis from sequencing performed at University of Ulm utilized Cologne Center for Genomics (CCG) Varbank pipeline v2.6 and user interface (Spielmann et al., *Genome Res* 2016 Feb; 26(2) 183-91.). Additional details of alignment and variant calling methods other than summarized above can be provided upon request.

Variants in EMC10 parents and affected individuals were confirmed by Sanger sequencing as indicated in Fig. 1a. See Table S1 for details of methodology applied to individual patient samples.

SNP-array genotyping

Genome-wide genotyping analysis was performed on Illumina Infinium Core-24 SNP arrays at the Yale Center for Genome Analysis (YCGA) (Family 1 (all living individuals), 2 (all living individuals), 3 (II-3, II-4, II-5, II-6, II-7, mother), and 6 (II-6 only)). Genotypes were called using GenomeStudio Software (Illumina). Low performing SNPs (those not called in all individuals in this study), low variance SNPs (those with minor allele frequencies (MAF) of 0 in all individuals in this study; that have an MAF < 5% documented in the Illumina Locus Report; or that deviate from HWE with P value < 0.00000001 among unrelated individuals run on the same chips), and SNPs with Mendelian and non-Mendelian errors (calculated with Merlin) were removed from the analysis using PLINK. PLINK was used to thin SNPs to reduce linkage disequilibrium. Merlin18 was used, with the trimmed SNP set, to calculate genome-wide multipoint LOD scores under a recessive mode of inheritance assuming a disease allele prevalence of 0.0001 and full penetrance. Single point linkage analysis was also performed using Merlin18 to the phenotype of intellectual disability against the EMC10 c.287delG mutation in all families where Sanger validation of the mutation was available.

For Family 5 and Family 6, Affymetrix genome-wide human SNP 6.0 arrays were performed on genomic DNA samples from all individuals. Autozygosity mapping was performed as previously described (Borck et al. *Am J Hum Genet* 2011).

Radiographic Evaluation

Original images from brain CT or brain MRI were obtained when available. Images were reviewed centrally by an experienced neuroradiologist for standardized assessment of imaging features. Similarly, original abdominal ultrasound images for affected individuals in Family 1, Family 6, and Family 7 were obtained and reviewed centrally by specialists in pediatric nephrology.

Blood RNA and DNA extraction

Blood from Family6: II-5 and unaffected mother Family6: I-2 were collected in Paxgene Blood RNA tubes. RNA was extracted using MagMAX for Stabilized Blood Tubes RNA (Thermo Fisher). DNA was extracted using the same protocol, replacing DNA digestion during extraction with 5ul RNAse A (NEB).

Generation of iPSC-derived neurons

Induced pluripotent stem cells (iPSC) were generated by Sendai-viral reprogramming as previously described (Schlaeger, et al., *Nat Biotechnol*. 2015 Jan:33(1) 58-63). Neurons were generated via NGN2-induced differentiation after stable integration of doxycycline-induced NGN2 expression vector. Cells were seeded in triplicate for biological replicated and NGN2 expression was induced by addition of doxycycline to culture media as previously described (Zhang, et al. *Neuron* 2013 June: 78(5) 785-98). Identity of iPSC lines were authenticated by STR profiling and lines were tested for mycoplasma.

RNA expression analysis by ddPCR:

Superscript IV VILO mastermix for reverse transcription was used to generate cDNA from 50ng RNA in triplicate from each sample. Mastermix without reverse transcriptase was used to confirm absence of DNA contamination. For RNA samples contaminated with DNA, an additional digestion with DNAse I (NEB) was performed prior to reverse transcription. 1:12 of total cDNA reaction (~4.2ng RNA equivalent) was used for ddPCR using two validated FAM dye-labeled Taqman probes (Probe 1 = Hs01041891_m1; Probe 2= Hs0382250_m1) targeting EMC10 or control VIC dye-labeled probe targeting ACTB (Hs01060665_g1). P-values were calculated by single-sided t-test.

Transfection of siRNA:

HEK293 cells were seeded at 150,000 cells per well and transfected 48 hours later using Lipofectamine 3000 Transfection reagent. 10ul of 20uM siRNA was transfected per well. SiRNA were purchased from ThermoFisher (Stealth siRNA, HSS154572, HSS154574, HSS178685). Lysates were collected 48 hours after transfection. Immunoblotting was performed using rabbit anti-human C19orf63/EMC10 (Abcam, ab185365) at 1:500 dilution. Knockdown in HEK293 was performed only once; this experiment has been performed in other cell lines (not shown) with similar results.

Protein Conservation analysis

Analysis of protein conservation was performed using ConSurf (Ashkenazy, et al. *Nucleic Acids Research* 2016), using a Bayesian model of best fit. Homologues were selected using default parameters: 3-iterations of CSI-BLAST from database Uniref-90, with maximum of 150 homologs and E-value cutoff of 0.0001.

Immunohistochemistry

Immunohistochemistry was performed on fixed and frozen human infant brain tissue (University of Maryland Brain and Tissue Bank of the NIH NeuroBioBank (sample number UMBN 5817)). Primary antibodies used were rabbit anti-human C19orf63/EMC10 (Abcam, ab185365), MAP2 (Abcam, ab5392), and mouse anti-human NeuN (Millipore, MAB377). DAPI staining was included in Fluoromount-G (Southern Biotech). Images were acquired on Zeiss LSM700 confocal microscope. See prior publication for details of protocol (Smith et al. *Neuron* 2018). This specimen staining included in the manuscript was performed one time; similar results were obtained in two other brain samples not shown.

Expression of mutant constructs and proteasomal inhibition

The open reading frame resulting from frameshift variant *EMC10*, from residue 1 to the terminal residue of p.Gly96ArgfsTer9, was cloned with a C-terminal V5 tag into pCMV plasmid (DNASU). For comparison, the first 103 amino acids of EMC10 ($EMC10_{1\text{-}103}$) and the EMC10 luminal domain ($EMC10_{1\text{-}221}$) were similarly cloned with C-terminal V5 tag. Plasmids were transiently transfected into HeLa cells (source of cells from Tian et al, Cell Rep 2019.). Cells were harvested 48 hours after transfection and lysed by RIPA buffer (50 mM Tris, pH 7.5, 1% NP-40, 150 mM NaCl, 0.5% sodium deoxycholate, 1% SDS, protease inhibitor cocktail).

Proteasomal inhibition was performed 48 hours after transfection using 25uM of MG-132 solubilized in DMSO for 1 hour or 4 hours (10mM stock, Millipore). An equal volume of DMSO was used as negative control for 4 hours. Cells were harvested as above at the indicated time points. Immunoblotting was performed using mouse anti-V5 antibody (Thermo, R960-25) at dilution 1:5000 and rabbit anti-GAPDH (Cell Signaling, 14C10) at dilution 1:10,000. Imaging was performed on LICOR Odyssey after staining with corresponding LICOR secondary antibodies.

<u>**Supplementary Figures**</u>

S1

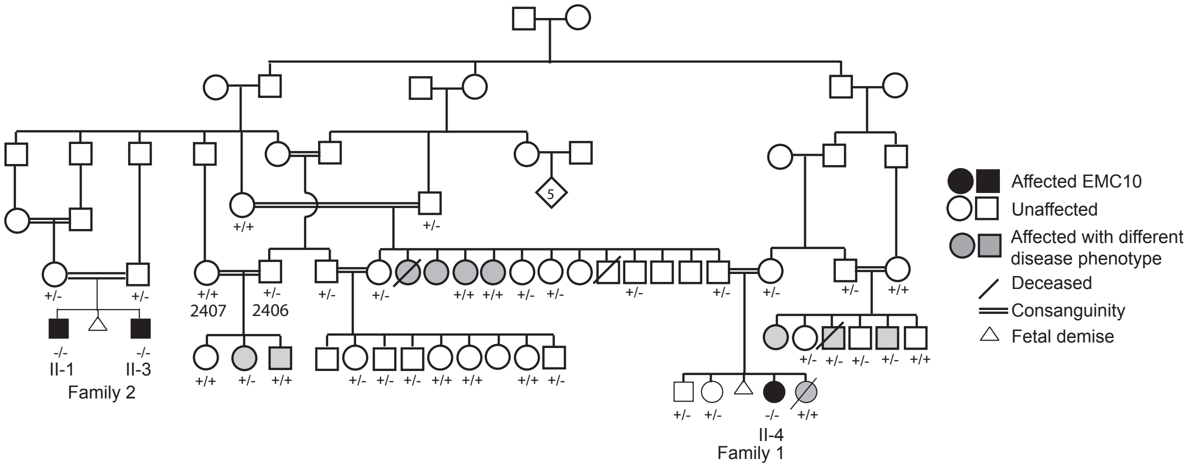

Figure S1. Expanded pedigree of relationship between Family 1 and Family 2. Sanger sequencing of *EMC10* was performed on numerous individuals, resulting *EMC10* genotypes are indicated as annotated. Individuals affected with a different disease phenotype not relevant to this manuscript are shaded grey.

S2

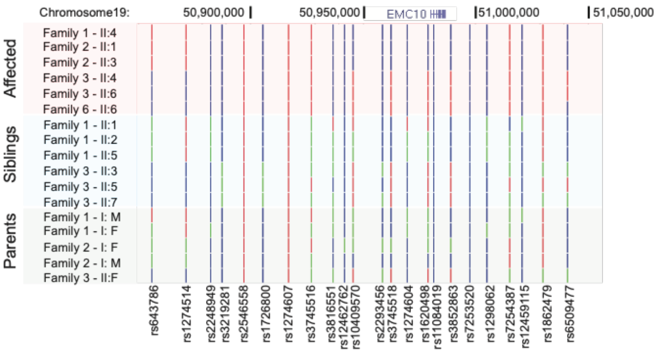

Figure S2. SNPs from Illumina Infininium Core-24 array for affected individuals, siblings, and parents near the *EMC10* locus. Homozygous SNPs are shown in red or blue. Heterozygous SNPs shown in green. Affected individuals in Families 1 and 2 share a haplotype; and a second distinct haplotype is shared by affected individuals in Family 3 and Family 6.

S3

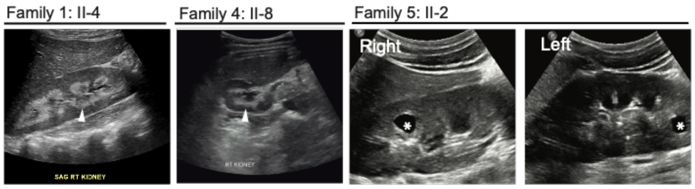

S4

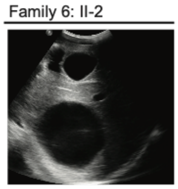

Figure S3. Transverse view of kidneys on renal ultrasounds showing medullary nephrocalcinosis (arrowheads) and bilateral renal cysts (asterisks).

Figure S4. Transverse ultrasound scan of the right hepatic lobe shows multiple hepatic cysts.

S5

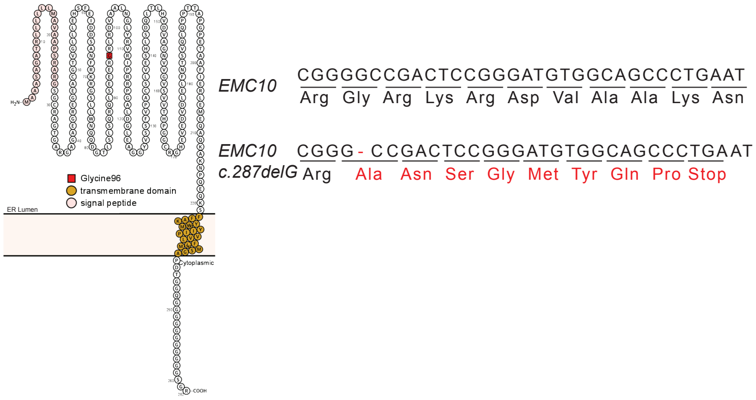

Figure S5. Coding sequence and amino acids changes in *EMC10* c.287delG (p.Gly96AlafsTer9).

S6

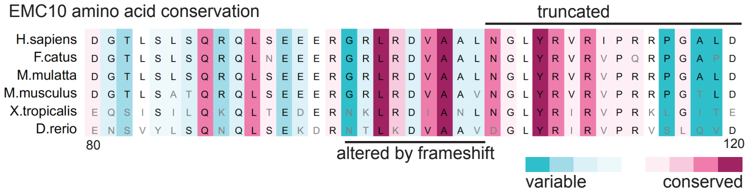

Figure S6. Amino acid conservation amongst all homologues of EMC10 at the region of the predicted EMC10 frameshift and truncation.

S7

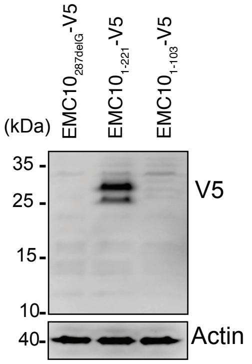

Figure S7. V5-tagged frameshift EMC10 ($EMC10_{287delG}$; based on *EMC10* c.287delG), EMC10 truncated at residue 103 ($EMC10_{1-103}$), or truncated after the entire lumenal domain at residue 221 ($EMC10_{1-221}$) were expressed in HeLa cells by transient transfection. Cell lysates were examined by immunoblot analysis detecting the V5 tag.

S8

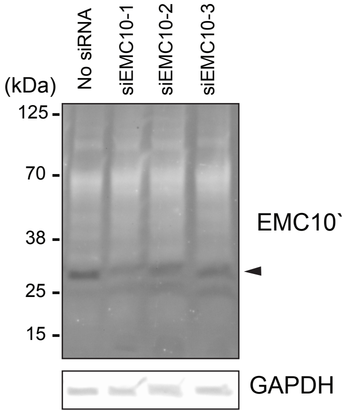

Figure S8. Assessment of specificity of anti-EMC10 antibody used in immunohistochemistry (Fig. 2g). HEK293 cells were transfected with three different siRNA targeting EMC10, and lysates were stained with the indicated antibody.

S9

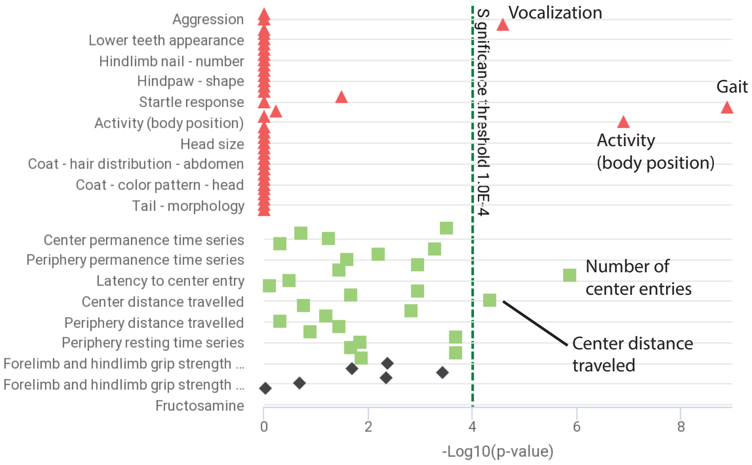

Figure S9. Phenotypes of *EMC10* knockout mice compared to control mice as characterized by the International Mouse Genotyping Consortium (www.mousephenotype.org). Each data point represents the p-value for a given phenotype. green = data from open field tests; red = SmithKline Beecham, Harwell, Imperial College, Royal London Hospital, phenotype assessment (SHIRPA) and dysmorphology; blue = grip strength.

Supplementary Tables

Table S1. Summary of clinical characteristics

Family	Family 1	Family 2		Family 3		Family 4	Family 5		Family 6				Family 7	SUMMARY
Gender	Female	Male	Male	Female	Male	Male	Male	Female	Male	Male	Male	Female	Female	
Age at evaluation	10 years	12 years	8 years	27 years	22 years	5 years	21 years	20 years	17 years	15 years	10 years	6y ears	3 months	
Origin	UAE	UAE	UAE	Saudi Arabia	Saudi Arabia	Saudi Arabia	Bedouin	Bedouin	Bedouin	Bedouin	Bedouin	Bedouin	Saudi Arabia	
Individual	II-4	II-1	II-3	II-4	II-6	II-8	II-1	II-2	II-1	II-2	II-5	II-6	II-1	
**GDD/ID**	+	+	+	+	+	+	+	+	+	+	+	+	N/A	12/13
**Abnormal social behavior**	+	+	+	-	+	+	+	+	+	+	+	+	N/A	11/13
**Seizures**	+	-	+	-	+	+	+	+	-	-	-	-	-	6/13
**Cubitus valgus**	-	-	-	-	-	-	+	+	+	+	+	+	-	6/13
**Hernia**	-	-	+	-	-	-	-	-	+	+	+	+	-	5/13
**Nephrocalcinosis**	+	+	-	+	**	-	-	-	-	-	-	-	+	≥4/13
**Polyhydramnios**	-	-	+	+	-	+	-	-	-	-	-	-	+	4/13
**Failure to thrive**	+	+	-	+	+	-	-	-	-	-	-	-	-	4/13
**Arachnodactyly**	-	-	+	+	+	-	-	-	-	-	-	-	-	3/13
**Ventricular septal defect**	-	-	-	-	-	-	-	+	+	-	-	-	-	2/13

nd = data not available; ** = unknown renal disorder, required renal transplantation;

GDD/ID = global developmental delay / intellectual disability; UAE = United Arab Emirates;

N/A=not applicable due to patient age (3 months) at time of ascertainment.

Table S2. Neurodevelopmental presentation and neuroimaging findings.

		Family 1	Family 2		Family 3		Family 4	Family 5		Family 6				Family 7
		II-4	II-1	II-3	II-4	II-6	II-8	II-1	II-2	II-1	II-2	II-5	II-6	II-1
Clinical presentation	**GDD/ID**													
	**Seizures**													
	**Attention-deficit / hyperactivity**													
	**Abnormal social skills**	shy				ag	ag	shy	shy	shy	shy		shy	
Neuroimaging	**Imaging Modality**	MRI		MRI	CT	CT	MRI	MRI	MRI	MRI	MRI	MRI	MRI	MRI
	**Thin corpus callosum**													
	**Cerebellar Tonsillar Ectopia or Chiari I**													
	**Myelination**	*											*	**
	**Ventriculomegaly**													
	**Grey Matter Heterotopia**													

red = abnormal findings; grey = data not available; ag=aggressive
*Central tegmental tract T2 prolongation
**= abnormal signal intensity on T1 and T2 sequences in the periatrial white matter consistent with perinatal event

Table S3. Renal presentation and imaging findings.

		Family 1	Family 2		Family 3		Family 4	Family 5		Family 6				Family 7
		II-4	II-1	II-3	II-4	II-6	II-8	II-1	II-2	II-1	II-2	II-5	II-6	II-1
Clinical Presentation	↑UOP	FP IP (red)		FP (red)			FP (red)							FP IP (red)
	ESRD					(red)								
Renal Ultrasound	Medullary Nephrocalcinosis	(red)	(red)	(grey)	(red)	(grey)								(red)
	Renal Cysts			(grey)	(red)	(grey)	(red)		(red)					
	CAKUT (Hydronephrosis or hydroureter)		(red)	(grey)		(grey)							(red)	

Red = abnormal findings; grey = data not available; FP = fetal polyhydramnios; IP = infantile polyuria; UOP = urine output; ESRD = end-stage renal disease; CAKUT = congenital anomalies of the kidney and urinary tract

Table S4. Comparison of EMC10 to EMC1 phenotype

	**Number of families affected**	
	**EMC1**	**EMC10**
Global developmental delay	4/4	6/6*
Seizures	1/4	5/7
Scoliosis	3/4	0/7
Head circumference	Zscore -4 to 0	Zscore -1.6 to +2
Truncal hypotonia	4/4	3/5*
Cerebellar atrophy	4/4	0/7
Cerebellar tonsilar ectopia / Arnold Chiari malformation	0/4	2/7
Cerebral atrophy	3/4	0/7
Foreshortened or atrophic corpus callosum	4/4	3/6*

Features are counted by family rather than by individual to provide consistency to published EMC1 phenotype as reported in Harel, et al. (*AJHG* 2016 Mar: 98(3) 562-70).
*Denominator adjusted to account for data that is not available from all families.

Table S5. EMC-dependent proteins with reported neurodevelopmental phenotypes

**Gene**	**Significant interaction in Tian, et al.**	**Significant interaction in Shurtleff, et al.**	**Gene Description**	**Disease Phenotype**	**Reference**
ATP6V0A1	+		V-type proton ATPase 116 kDa subunit a isoform 1	Implicated in Rett-like syndrome	Iwama, et al. J Med Genet 2019; 56: 396-407.
SLC9A7	+		Sodium/hydrogen exchanger 7	Mental retardation, X-linked 108	OMIM Phenotype 301024
ATP6V0C	+		V-type proton ATPase 16 kDa proteolipid subunit	One of 3 genes in microdeletion syndrome - microcephaly, epilepsy, developmental delay	Mucha, et al. Genetics in Medicine 2018; 21(5): 1058-1064
ITPR1	+		Inositol 1,4,5-trisphosphate receptor type 1	Gillespie syndrome; Spinocerebellar ataxia 15; Spinocerebellar ataxia 29	OMIM Phenotypes 206700; 606658; 117360
TMEM199	+		Transmembrane protein 199	Congenital disorder of glycosylation, type IIp	OMIM Phenotype 616829
FDFT1	+	+	Squalene synthase	Seizures, ID, brain white matter loss, FTT	OMIM Phenotype 184420
BCAP31	+	+	B-cell receptor-associated protein 31	X-Linked Phenotype with Deafness, Dystonia, and Central Hypomyelination	OMIM Phenotype 300475
SLC1A3/ GLAST		+	Excitatory amino acid transporter 1	Episodic ataxia type 6	OMIM Phenotype 600111
TREX1		+	Three-prime repair exonuclease 1	Aicardi-Goutierres Syndrome 1	OMIM Phenotype 225750
CTSA		+	Lysosomal protective protein	Galactosialidosis	OMIM Phenotype 256540

Sixty-one high-confidence EMC-dependent proteins based on publications Tian et al. (*Cell Rep* 2019) and Shurtleff, et al. (*Elife* 2018) were reviewed in PubMed or through Online Mendelian Inheritance in Man (OMIM; https://omim.org).

Table S6. Genomic techniques used to assess affected individuals

Subject	WES (BGI/ Broad Institute)	WES (Yale)	WES (Ulm)	WES (Centogene)	WGS (Centogene)	Sanger Confirmation	SNP Array
Family 1: II-4	N	Y	N	N	Y	Y	Y
Family 2: II-1	N	N	N	N	Y	Y	Y
Family 2: II-3	N	N	N	N	Y	Y	Y
Family 3: II-4	Y	N	N	Y	N	Y	Y
Family 3: II-6	Y	N	N	Y	N	Y	Y
Family 4: II-8	N	N	N	N	Y	Y	N
Family 5: II-1	N	N	N	N	N	Y	N*
Family 5: II-2	N	N	N	N	N	Y	N*
Family 6: II-1	N	N	Y	N	N	Y	N*
Family 6: II-2	N	N	N	N	N	Y	N*
Family 6: II-5	N	N	N	N	N	Y	N*
Family 6: II-6	N	N	N	N	N	Y	Y
Family 7: II-1	N	N	N	Y	N	Y	N

Y=yes; N=no; *SNP array performed for initial candidate gene discovery, data not available / not included in this publication

Table S7. Summary statistics for genome and exome sequencing.

Individual	Type	Site	Mean coverage (depth)	Percent base pairs covered		
				10x	20x	50x
Family 1: II-4	WGS	Centogene	35	98.51	96.16	2.89
Family 1: II-4	WES	Yale Univ.	45	95.32	89.00	36.92
Family 2: II-1	WGS	Centogene	28	98.2	84.91	0.76
Family 2: II-3	WGS	Centogene	41	99.23	96.24	14.7
Family 3: II-4	WES	Broad/BGI	90	72.49	65.57	49.06
Family 3: II-6	WES	Broad/BGI	65	73.16	65.15	42.81
Family 3: II-6	WES	Centogene	108	98.45	94.92	76.6
Family 3: II-4	WES	Centogene	105	98.15	94.6	76.25
Family 4: II-8	WGS	Centogene	57	98.9	98.73	67.83
Family 6: II-1	WES	Univ. of Ulm	Metrics not available			
Family 7: II-1	WES	Centogene	104	99.68	99.49	93.72
